# Therapeutic PCL scaffold for reparation of resected osteosarcoma defect

**DOI:** 10.1038/s41598-017-12824-3

**Published:** 2017-10-04

**Authors:** Ilaria E. Palamà, Valentina Arcadio, Stefania D’Amone, Mariano Biasiucci, Giuseppe Gigli, Barbara Cortese

**Affiliations:** 1 Nanotechnology Institute, CNR-NANOTEC, via Monteroni, Lecce, 73100 Italy; 2 Nanotechnology Institute, CNR-NANOTEC, University La Sapienza, P.zle A. Moro, Roma, 00185 Italy; 3 Center for Life Nano Science@Sapienza, Istituto Italiano di Tecnologia,Viale Regina Elena 291, 00161 Roma, Italy; 4 Department Matematica e Fisica ‘Ennio De Giorgi’, University of Salento, via Monteroni, Lecce, 73100 Italy

## Abstract

Osteosarcomas are highly malignant tumors, which develop rapid growth and local infiltration, inducing metastases that spread primarily in the lung. Treatment of these tumors is mainly based on pre- and post-operative chemotherapy and surgery of the primary tumor. Surgical resection though, generates bone defects. Reparation of these weaknesses presents formidable challenges to orthopedic surgery. Medicine regenerative grafts that act as both tumor therapy with constant local drug delivery and tissue regeneration may provide a new prospect to address this need. These implants can provide sustained drug release at the cancer area, decreasing systemic second effects such as inflammation, and a filling of the resected tissues with regenerative biomaterials. In this study microporous poly-ε-caprolactone (PCL) scaffolds have been developed for sustained local release of anti-inflammatory drug dexamethasone (DXM), used as drug model, in cancer medicine regenerative field. The microporous PCL matrix of the scaffolds supported the attachment, proliferation and osteogenic differentiation of osteoblast-like cells, while the polyelectrolyte multilayers, anchored to the inner pore surfaces, sustained locally DXM release. These microporous scaffolds demonstrate the ability to deliver DXM as a localized tumor therapy and to promote proliferation and differentiation of osteoblast-like cells *in vitro*.

## Introduction

Osteosarcomas are highly aggressive tumors with a high prevalence in pediatric patients. The chance of recovery and choice of treatment depend upon many factors such as the size and stage of the tumor, aggressiveness and location^1^. Until a few decades ago, osteosarcomas’ therapy involved invasive surgery, with inevitable drawbacks such as frequent recurrences as well as risk of major impacts on the psycho-mental health of the cancer patient^2^. Today, the therapy is more conservative through administration of chemotherapeutic drugs before and after surgery, with the aim to reduce the tumor mass before cancer resection and subsequently eliminating any residual tumor cells^3,4^. However, osteosarcoma resection determines large bone defects which regenerative repair currently represents a challenging issue during surgery with side effects on normal tissues. Therefore, there is considerable interest in developing new therapeutic strategies for the design and engineering of bone substitutes^5^. Recent advances in the field of bone tissue engineering has focused on the realization of three dimensional (3D) porous scaffolds which can mimic the architecture and the mechanical and physical properties of native bone, providing support and facilitating functional cell processes, critical for tissue regeneration^6,7^. Numerous studies on porous scaffolds have been reported showing effective capability of supporting cellular interactions, preserving tissue volume, and delivering biological agents^7–9^. An increasing volume of work based on bioceramics and biodegradable polymers and their composites has been investigated^10,11^. However drug release patterns are difficult to control in bioactive ceramic scaffolds^12^. A higher local control of drug delivery was obtained with synthetic biodegradable polymeric materials such as poly(lactic-co-glycolic acid) (PLGA)^13^ and poly(propylene glycol-fumerate)/ methylmethacrylate^14^; nevertheless, reduced osteoconduction was observed with adverse tissue response owing to inflammation due to acidic degradation^15^. Within the class of biodegradable polymers, poly (є-caprolactone) (PCL) has drawn a great deal of attention owing to its biocompatibility, biodegradability, structural stability and mechanical properties. Hu and colleagues developed nanocomposite poly(L-lactic acid)-modified hydroxyapatite (g-HAp)–poly(ε-caprolactone) (PCL) porous scaffolds, by solvent evaporation based on water-in-dichloromethane (W/O), using ibuprofen (IBU) as a model drug. They showed good adhesion and proliferation of mouse bone mesenchymal stem cells which indicated an effective biocompatibility^16,17^. Rapid proliferation and oriented migration of human bone marrow stromal cells and trabecular osteoblasts were shown on porous PCL/poly-L-lactide (PLLA) fibrous composite scaffolds^18,19^. More recently, several studies have developed composite scaffolds using PCL and Chitosan. PCL scaffolds embedded with a porous matrix composed of chitosan, nanoclay, and β-tricalcium phosphate showed high cell viability and growth, with good cell infiltration to the pores of the scaffold and local sustained release of an anticancer drug *in vitro* ^20–22^. However some were physically unstable because of the lack of structure integrity of the scaffolds^23^.

A dual therapy approach through embodiment of an anti-cancer drug into a biodegradable tissue-regenerative scaffold would represent a key aim in biomedical science nowadays as it would aid to inhibit tumor cell proliferation while the scaffold fills the defect after tumor resection supporting local tissue regeneration of within a controlled microenvironment^9,10,24,25^. In fact, the incorporation of drug delivery systems, such as nanoparticles, in scaffolds can improve the therapeutic action and safety of bioactive molecules, delivering them at the exact action site by a controlled, sustained and local release^24,26^. Various systems for local drug delivery have showed good release of drug active against cancer cells^27–30^. However, despite their considerable promise of regenerative drug-based scaffolds, it is very problematic to control the drug quantity and release kinetic in those systems^11^. Moreover, to the best of the present authors’ knowledge, there has not been any approach in which nanoscaled polyelectrolyte multilayers embedded in a poly-ε-caprolactone (PCL) scaffold has been employed as a drug delivery system. Herein, we report the design and *in vitro* validation of porous PCL scaffolds for the regeneration of bone tissue. The scaffold was obtained by combining two components: a PCL matrix for cell growth support and a multilayer polyelectrolyte for sustained local drug delivery. Porous PCL scaffolds were prepared using a solvent casting and particulate leaching technique. Nanoscaled polyelectrolyte multilayers loaded with dexamethasone (DXM), a clinical model synthetic glucocorticoid with potent anti-inflammatory and immunosuppressive action^31^, were incorporated into the PCL scaffold through CaCO_3_ microparticles, used as porogen agent. The advantage of using CaCO_3_ microparticles covered with multilayer polyelectrolyte as a system for drug delivery, instead of whole nanoparticles had a dual purpose: 1) to create porosity in the scaffolds and 2) to allow a controlled and localized, easy *in situ* release of the drug associated with polyelectrolyte multilayers. The structure and composition of the PCL scaffolds were characterized by optical microscopy and scanning electron microscopy (SEM). We then evaluated the effects of drug loading (10 or 100 nm per composite formulation) on the scaffold properties and release kinetics of a model drug. Furthermore, the therapeutic capacity of PCL scaffolds was evaluated by measuring the viability of human osteosarcoma derived osteoblasts (cell line MG63) cells *in vitro*. Finally, the biocompatibility and bone-forming ability of our 3D microporous PCL scaffolds were investigated by culturing MG63 *in vitro*, ensuring a good environment for cell proliferation and osteogenic differentiation with an ability to load and release therapeutic agent in a sustained manner.

## Results and Discussion

Cancer local relapse can be attributed to insufficient resection of cancer, hidden multifocal cancer, and absence of post-surgical radiation therapy^32^. Furthermore, local relapse has been shown to be present at the primary place of the primary cancer and at the primary site of therapeutic failure and inflammation^33^. We aimed to address this important issue, in the present study, by developing a dual therapy drug delivery system. In particular, our scaffolds present two main components: (1) a biodegradable microporous structure aimed to mimic the complex bone morphology and its structure for cell growth/proliferation support and (2) to introduce a sustained local drug delivery system. In fact, while the therapeutic active agent inhibits cancer cell proliferation or reduces the inflammation process, the scaffold allows the filling of the defect after cancer resection, supporting the local tissue regeneration.

### Synthesis and characterization of the porous scaffold

**Figure 1:**
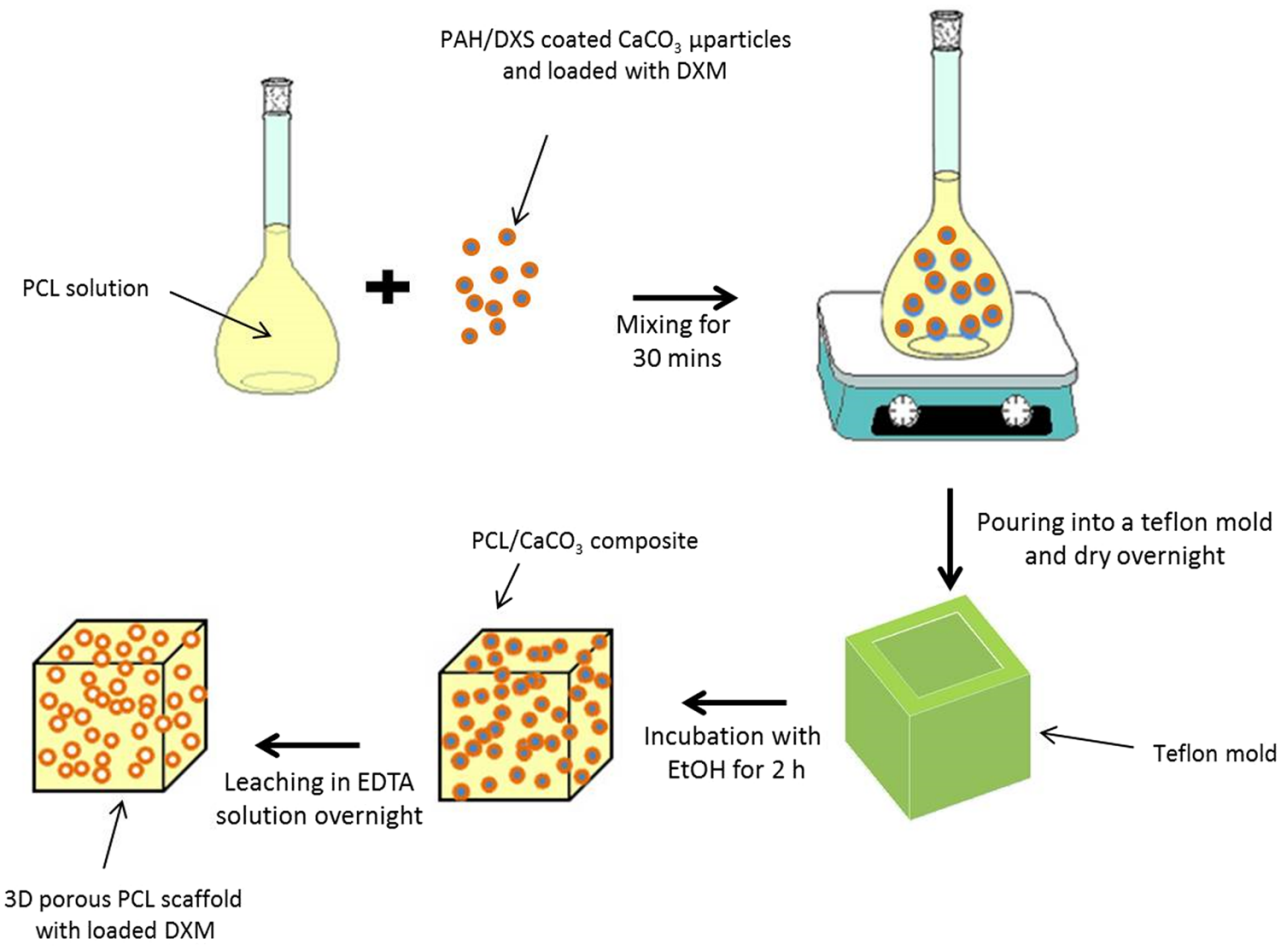
A schematic illustration of 3D microporous PCL scaffolds engineered for sustained release of DXM.

3D porous scaffolds were prepared to mimic the native bone micro-nanostructure. Synthesis of bifunctional 3D microporous PCL scaffolds engineered with polyelectrolyte multilayers as local drug delivery systems were successfully obtained, Fig. 1.

**Figure 2:**
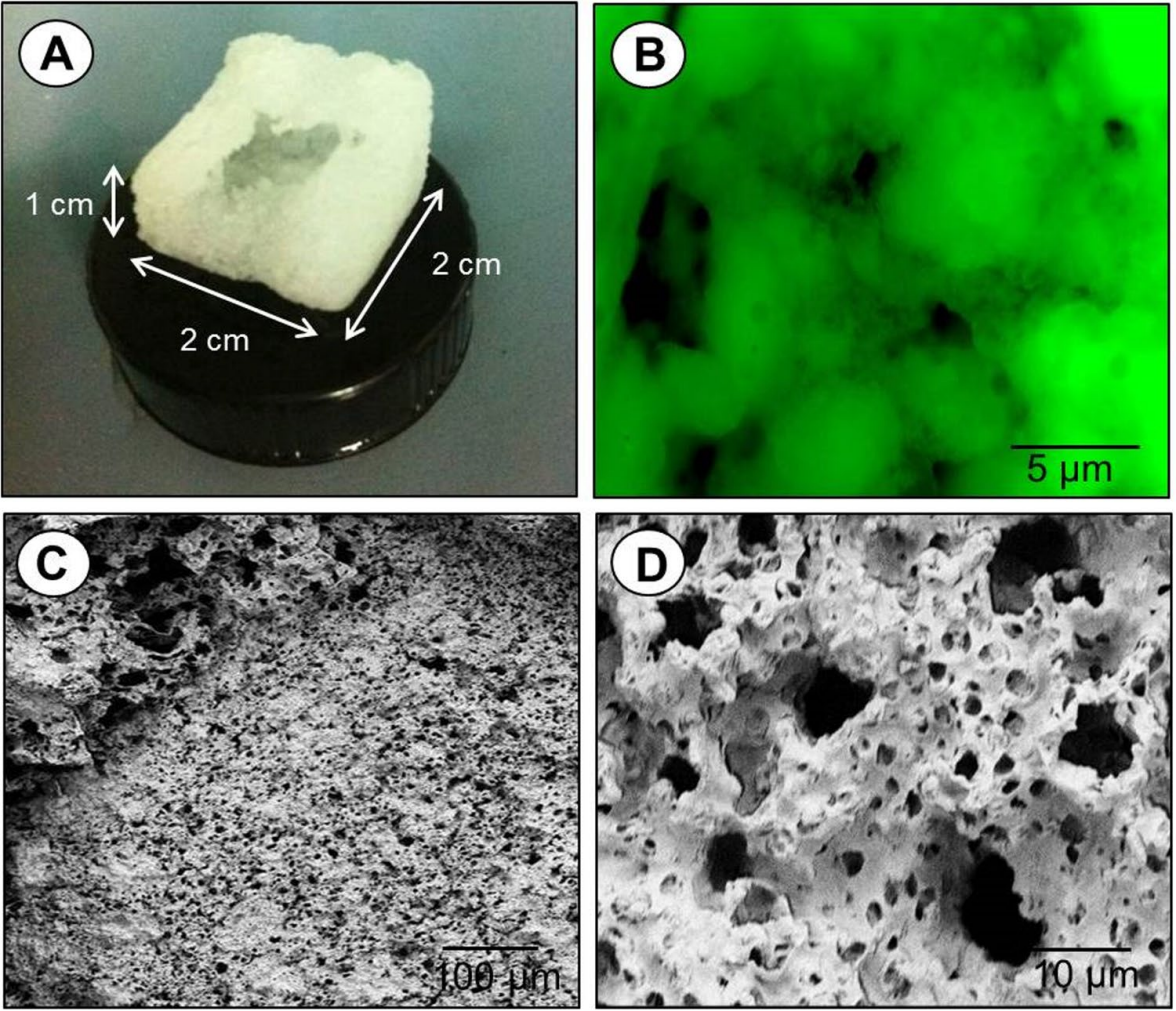
Photography (**A**), fluorescent image (**B**) and SEM images (**C**,**D**) of 3D microporous PCL scaffold. *Scale bars*: (**B**) *5* *µm*, (**C**) *100* *µm* and (**D**) *10* *µm*.

Scaffolds (Fig. 2) were obtained using solvent casting and particulate leaching (SC/PL). Scaffolds should offer a microenvironment which facilitates cell migration and in which the passage of nutrients, O_2_ and metabolic waste products are allowed. As observed, the PCL scaffolds exhibited a highly open-porous structure with interconnected porous walls. The scaffold microporosity was obtained using CaCO3 microparticles as biocompatible porogen agent. A solution of PCL in chloroform was mixed with coated CaCO_3_ microparticles, poured into a mold and dried overnight. In this way, a PCL/coated CaCO_3_ composite was obtained. CaCO_3_ microparticles, with an average diameter of 1–5 µm^34^, have shown no cytotoxicity and can be easily removed with a non-toxic ethylenediaminetetraacetic acid (EDTA) solution^35^.

The leaching in an EDTA solution of the CaCO_3_ core allowed to create the voids in the scaffold, Fig. 2B. In fact, the CaCO_3_ microparticles can be easily dissolved in a non toxic aqueous solution as the outer layer of polyelectrolytes on the coated CaCO_3_ microparticles exhibit a positive charge (+10 mV ± 0.25 mV) while the PCL polymer has a negative charge (−15 mV ± 0.09 mV) as measured through DLS analysis. These opposite charges allow an electrostatic interaction of polyelectrolyte multilayers with the PCL. CaCO_3_ microparticles were coated with the polyelectrolyte multilayers using poly (allylamine hydrochloride)/dextran sulfate sodium salt (PAH/DXS), loaded with DXM, hence creating an immunopriviledged environment. CaCO_3_ microparticles coated with PAH/DXS polyelectrolyte multilayers, with an average diameter of 1–5 µm, have shown a high encapsulation efficiency (84.3 ± 1.65%) of DXM (final concentration ranging from 10 to 100 μM) and a good stability under physiological conditions^36,37^.

The microporosity of our PCL scaffold was assessed by scanning electron microscopy (Fig. 2C,D). The enlarged view (Fig. 2C) indicates a heterogeneous microporosity and the globular-shaped structures (Fig. 2D) improved the surface roughness and surface area.

Surface stiffness can modulate cell interaction with the substrate, representing an important aspect for osteoblast differentiation^38–42^. Ideal elastic moduli of a biomaterial must be akin to *in vivo* tissue, in the range of MPa - GPa^43,44^. In literature, Young Modulus (YM) of PCL is very different and depends on manufacturing (electrospun fibers or scaffolds). For example, PCL electrospun nanofibers typically display YM below 300 MPa and 3 GPa^45^. On the other hand, the YM of PCL scaffolds present different values depending on the synthesis method and degree of porosity, for example, PCL scaffolds produced by Selective Laser Sintering^46^ displayed a YM between 16.1 MPa and 343.9-363.4 MPa, these values depending upon the building orientation.

**Figure 3:**
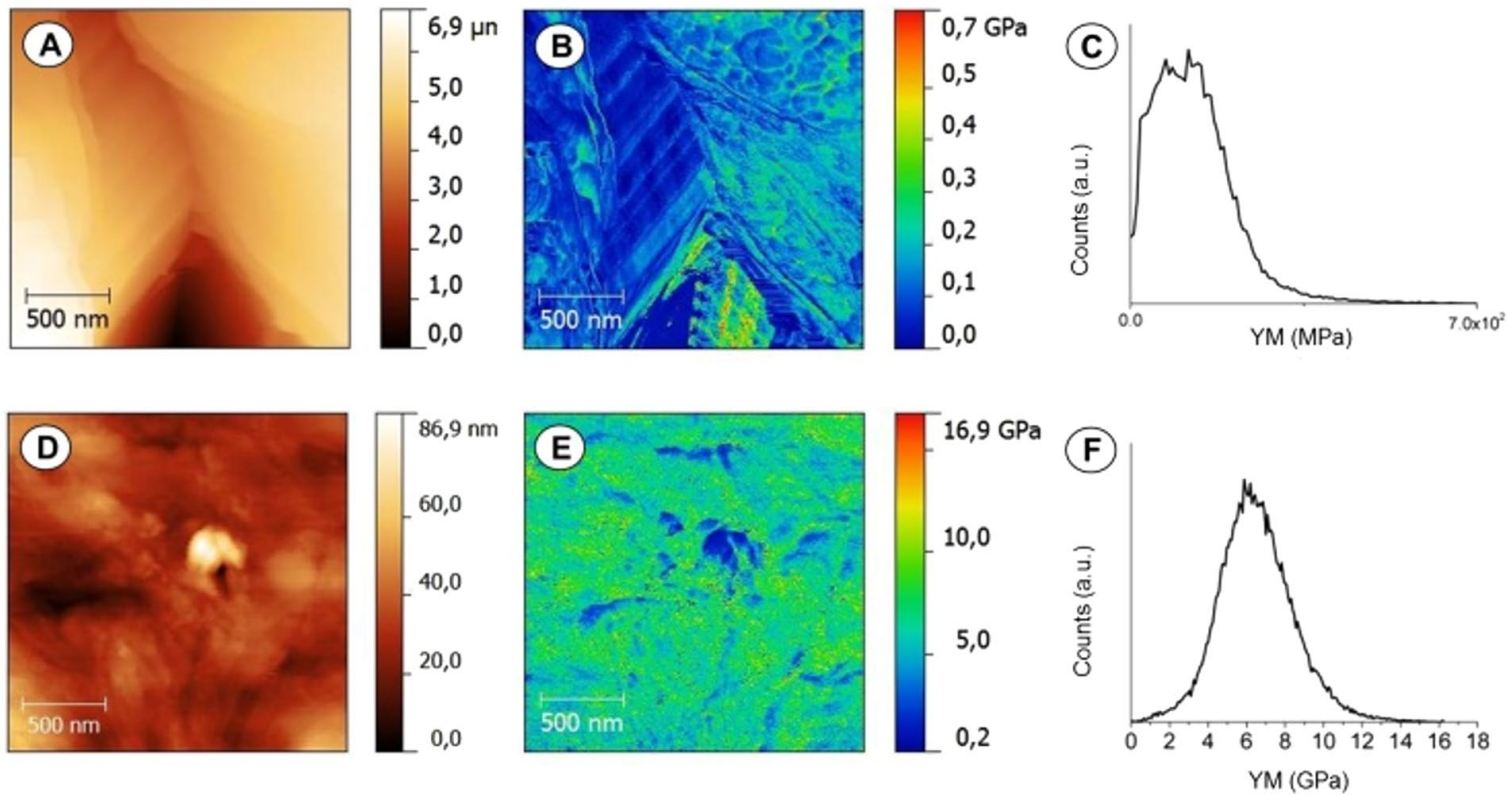
AFM images (**A**,**D**) and Derjaguin, Muller, Toropov (DMT) map of the Young’s modulus (**B**,**E**) of microporous PCL scaffold (**A**,**B**) and non-porous PCL scaffold (**D**,**E**). The DMT map (**B**,**E**) reports the Young’s modulus (YM) calculated on microporous PCL scaffolds (**C**) and non-porous PCL scaffold (**F**). A representative result of three independent experiments is shown. *Scale bars: 500 nm*.

The surface mechanical properties of our non-porous and microporous PCL scaffolds were investigated through AFM probe indentation technique. The value of the surface Young’s modulus is shown in Fig. 3. In particular, the two scaffolds drastically differed in Young’s modulus of a hundred-fold range. Specifically, the Young’s modulus of non-porous PCL scaffolds was 10 GPa ± 0.25 GPa, 100-fold higher than the microporous PCL scaffolds (0.1 GPa ± 0.05 GPa), Fig. 3C,F.

The non-porous PCL scaffolds were obtained by casting the PCL polymer in a Teflon mould, therefore the bulk characteristics differ from a porous or fibre structure, and consequently we measured a YM of 10 GPa. Indeed, the voids present in the microporous PCL scaffolds decreased considerably the stiffness, which can be modulated changing the ratio between PCL polymer and CaCO_3_ microparticles. In this way, we can obtain microporous scaffolds for application in different regenerative medicine fields, such as bone, vessels, liver, etc.

The surface wettability of the scaffolds was evaluated by water contact angle. Surface hydrophobicity represents, in fact, a crucial factor for cell adhesion, proliferation and differentiation on biomaterials^47,48^, The hydration degree of porous PCL scaffolds ranged from 25.14% ± 0.81 to 39.05% ± 0.57 (Figure S1 of the supplementary information) and remained constant up to 96 hours indicating that porous scaffolds were entirely hydrated. On the contrary, the non-porous scaffolds were hydrophobic. The CA of non-porous PCL scaffold was 68.36° ± 0.47°, while the CA of microporous PCL scaffold was 86.33° ± 0.65°. The discrepancy in the CA values was associated to the microporosity of the different surfaces. More specifically the porous architecture within the PCL structure creates air pockets, which influence the water contact angle and prevents the drop to spread within the structure. The higher experimental contact angle therefore originates from air pockets trapped between solid and liquid surface due to roughness.

### Protein adsorption to microporous and non-porous PCL scaffolds

Several proteins, such as immunoglobulins, fibronectin, etc. adsorb onto biomaterial surfaces when in contact with physiological fluids and modulate subsequent cellular behaviours. Cell adhesion to biomaterial surface need stable contact sites^49^, that are permitted thanks to the adsorption of serum and ECM proteins onto the material surface. Therefore, adsorbed proteins act as a key role in determining the cell interaction nature with the biomaterials.

**Figure 4:**
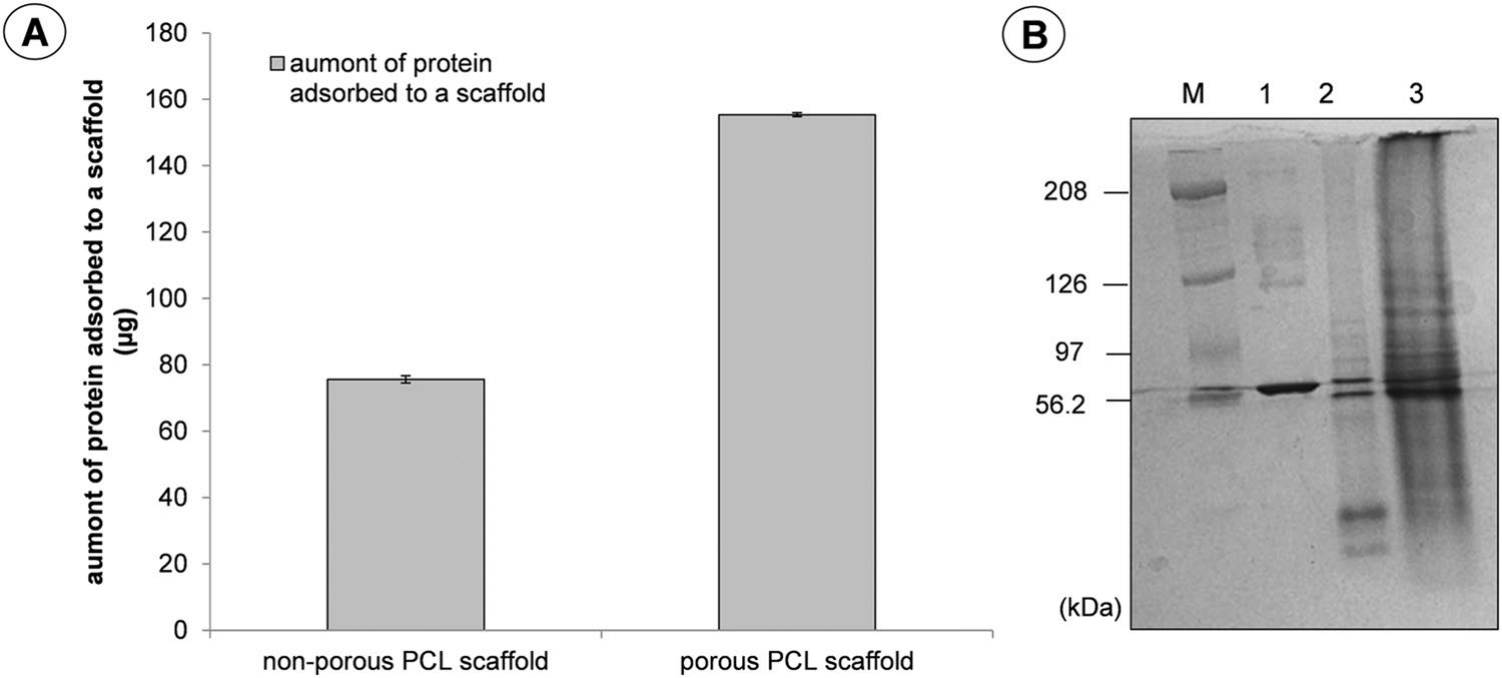
(**A**) Amount of adsorbed serum proteins to the porous and non-porous PCL scaffolds. Representative measurements of three distinct sets of data have been reported with no significant difference between values among scaffold groups (*t-Student’s test*, *P* < *0*.*05*). (**B**) SDS-polyacrylamide gel stained with Coomassie brillant blue, Image cropped from Figure S3. Lane M, high molecular weight marker; Lane 1, bovine serum proteins; Lane 2, adsorbed bovine serum proteins to the non-porous PCL scaffold; Lane 3, adsorbed bovine serum proteins to the porous PCL scaffold. A representative result of three independent experiments is shown.

Microporous and non-porous PCL scaffolds were incubated in foetal bovine serum. As shown in Fig. 4A, the microporous PCL scaffold adsorbed 2-fold higher quantity of serum proteins than the non-porous PCL scaffolds. Evidence was confirmed by SDS-polyacrylamide gel electrophoresis (SDS-PAGE) analysis (Fig. 4B), showing that major amounts of serum proteins were adsorbed to the microporous PCL scaffold (Fig. 4B, lane 3). Also, the outline of adsorbed proteins to the microporous PCL scaffold was different from that to the non-porous PCL scaffold (see Fig. 4B, lane 2,3), showing that microporous scaffolds improve protein adsorption, contributing to cell attachment.

### *In vitro* drug release from microporous PCL scaffold

**Figure 5:**
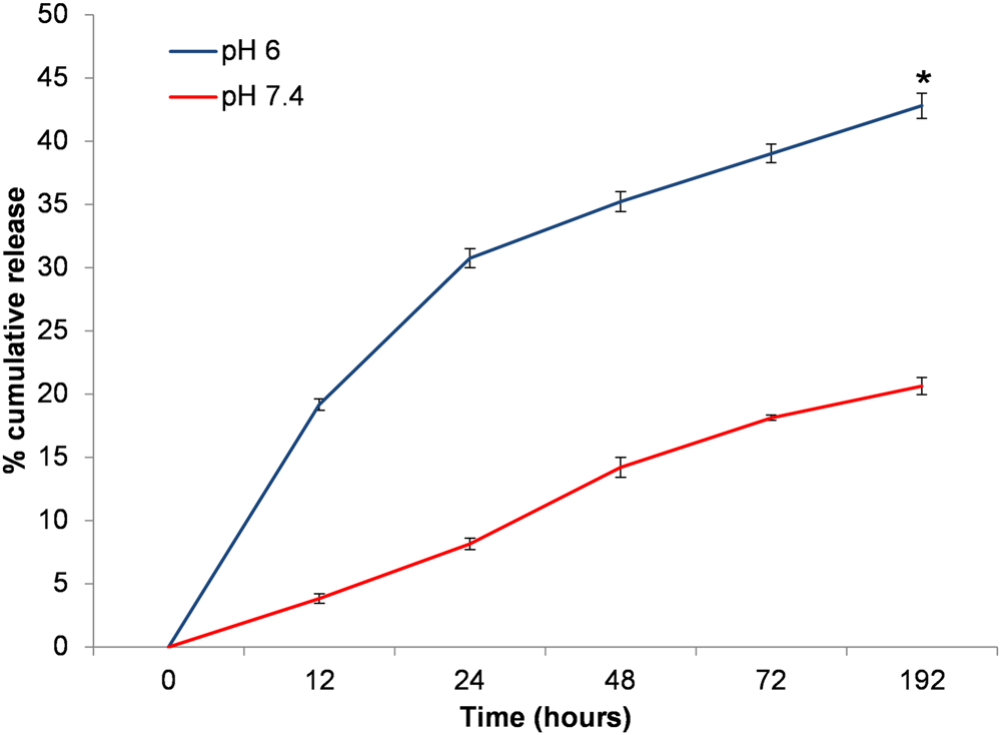
*In vitro* DXM (final concentration 10 nM) cumulative release from porous PCL scaffolds at neutral condition (pH 7.4) and acidic conditions (pH 6.0) at 37 °C. Representative measurements of three distinct sets of data have been reported, *indicates *P*-values of <*0.05* for *Student’s t-test*.

To ensure that the polyelectrolyte multilayers anchored on the holes’ inner surfaces of microporous PCL scaffolds released the encapsulated DXM in a controlled manner, we have assessed the dexamethasone (DXM) release from microporous PCL scaffolds in different conditions. Dexamethasone was used as a model drug to validate the drug release kinetic from the scaffold. As in pathological conditions such as inflammation or cancer, different pH between physiological (neutral pH) and pathological (acid pH) environments are present, we have observed that the drug release from the microporous PCL scaffolds was governed by medium pH and release time (see Fig. 5). The % cumulative DXM release was much higher in the presence of acid pH than physiological conditions alone. For example, DXM release at pH 7.4 was slow, with release percentage of about 20.65% ± 0.67% in 192 hours. Whereas, at pH 6.0, drug release rate was faster, with about 42.79% ± 0.98% of the DXM released.

The increased drug release to pH 6.0 is probably associated with polymer hydrolysis under acid situations, while the low rate of drug release to physiological pH can be associated with the drug passive diffusion. It can be presumed that under physiological conditions, more drug molecules remain for longer time encapsulated in the scaffolds. On the contrary, with lower pH, a more rapid release can be observed, improving in this way the cancer therapy efficacy.

### *In vitro* biological evaluation

**Figure 6:**
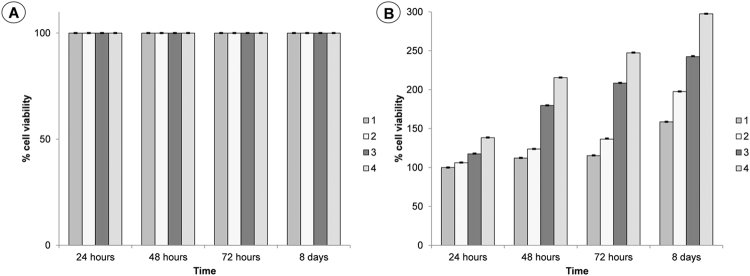
(**A**) Trypan blue test and (**B**) MTT assay for cellular viability of MG63 osteoblast cells cultured for 1, 2, 3, 8 days on non-porous PCL scaffold (1), porous PCL scaffold without loaded DXM (2), porous PCL scaffold with loaded DXM to final concentration of 10 nM (3) and porous PCL scaffold with loaded DXM to final concentration of 100 nM (4). Representative measurements of three distinct sets of data have been reported and no significant difference between values at different time points (*t-Student’s test*, *P* < *0.05*).

For a clinical achievement of a scaffold, it’s important that the scaffold does not induce adverse effects and promotes a strong cell attachment, as well as proliferation and differentiation. The adhesion and proliferation of MG63 osteoblast-like cells on the non-porous and microporous PCL scaffolds with/without loaded DXM (final concentration of 10 nM and 100 nM) were evaluated. Viability and proliferation of the MG63 osteoblast-like cells were assessed using a Trypan Blue exclusion test and a MTT assay, respectively. In terms of cell viability (Fig. 6), MG63 osteoblasts cultured on all samples displayed a significantly high viability. Whereas, in terms of cell proliferation (Fig. 6B), osteoblasts seeded on microporous PCL scaffold loaded with DXM of different concentrations, displayed significantly a dose-dependent proliferative effect. Osteoblasts seeded on non-porous, microporous PCL scaffolds without loaded DXM showed a kinetic of proliferation much slower with respect to the osteoblasts cultured on DXM loaded scaffolds.

**Figure 7:**
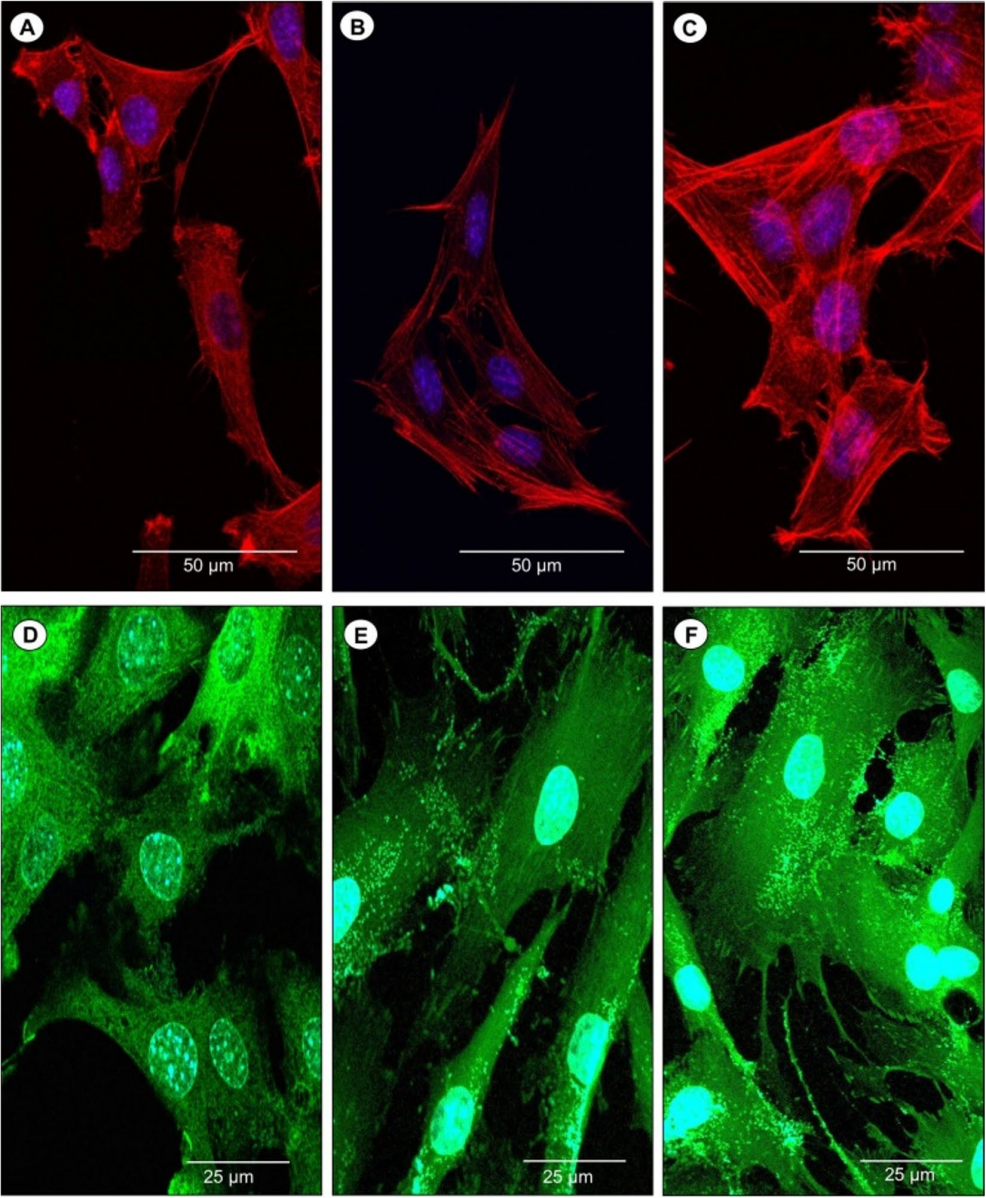
Confocal laser scanning images of MG63 osteoblasts seeded on non-porous PCL scaffolds (**A**,**D**), microporous PCL scaffold without (**B**,**E**) or with (**C**,**F**) loaded DXM (final concentration 10 nM) for 24 hours and stained for actin (red) or vinculin (green). Nuclei stained with DAPI (blue). A representative result of three independent experiments is shown. *Scale bars*: (**A**,**B**,**C**) *50* *µm* and (**D**,**E**,**F**) *25* *µm*.

After 1 day in culture, we found that cells showed good attachment and spreading on the non-porous, microporous PCL scaffolds without loaded DXM and microporous PCL scaffolds with loaded DXM to final concentration of 10 nM (Fig. 7). The spreading and elongated morphology of osteoblasts seeded on microporous PCL scaffolds with or without loaded DXM (Fig. 7B,C) confirmed that the cells were firmly anchored to the surface scaffolds. In particular, their elongated fibroblast-like shapes were characterized by increased and more sophisticated actin branching structures with polarized filopodia protrusions, compared to the same cells seeded on non-porous PCL scaffolds (Fig. 7A). In addition, we have analysed the distribution of vinculin. A random spotted distribution of vinculin was observed in MG63 osteoblasts seeded on surface of non-porous PCL scaffolds (Fig. 7D), whereas a cortical and homogenous cytosolic staining of vinculin was evident in the same cells plated on microporous PCL scaffolds without or with loaded DXM (Fig. 7E,F).

**Figure 8:**
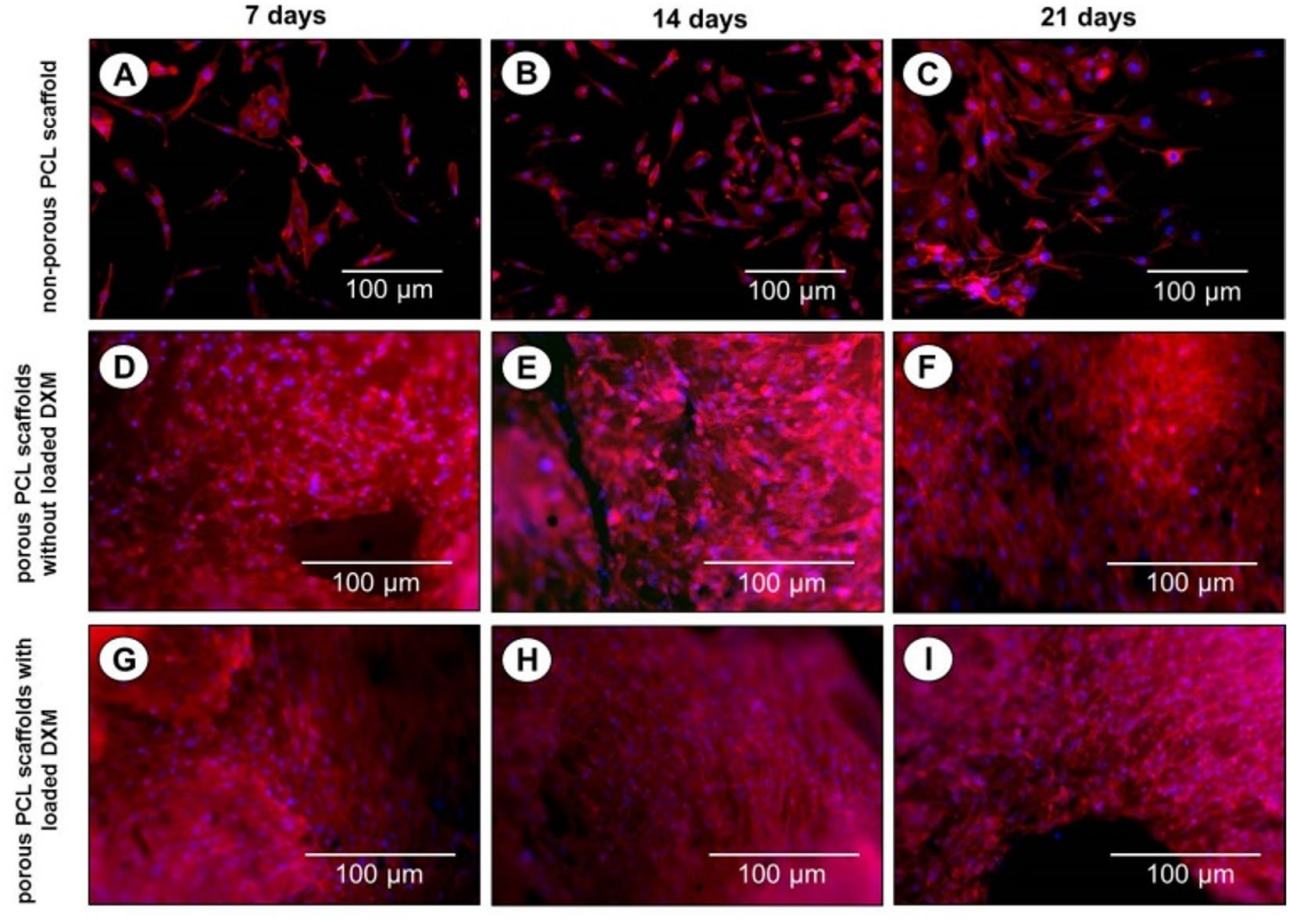
Confocal laser scanning images of MG63 osteoblasts seeded on non-porous PCL scaffolds (**A**,**B**,**C**), microporous PCL scaffold without (**D**,**E**,**F**) or with (**G**,**H**,**I**) loaded DXM (final concentration 10 nM) for 7,14,21 days and stained for actin (red). Nuclei stained with DAPI (blue). A representative result of three independent experiments is shown. *Scale bars: 100* *µm*.

On non-porous PCL scaffolds (Fig. 8A,B,C and Figure S2A of the supplementary information), a poor colonization was observed. On the other hand, a high degree of colonization and proliferation of microporous PCL scaffolds with loaded DXM (final concentration of 10 nM) by MG63 osteoblasts was perceived (Fig. 8G,H,I and Figure S2C of the supplementary information) after 7, 14 and 21 days of culture. A good colonization of microporous PCL scaffolds without loaded DXM, is evident by the confocal images showed in Fig. 8D,E,F and Figure S2B of the supplementary information.

**Figure 9:**
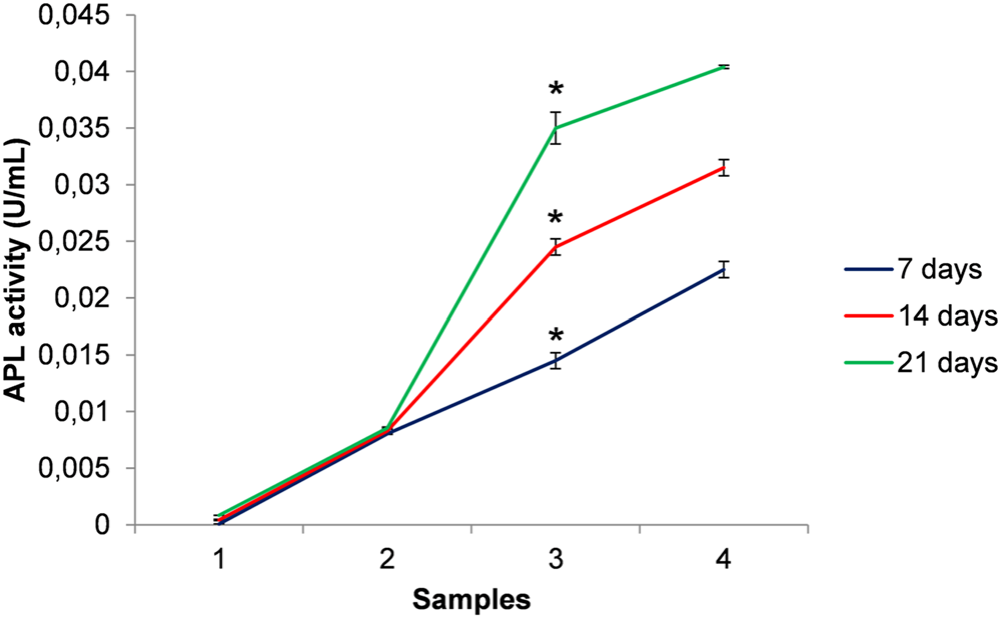
ALP activity for cellular viability of MG63 osteoblast cells cultured for 7,14,21 days on non-porous PCL scaffold (1), porous PCL scaffold without loaded DXM (2), porous PCL scaffold with loaded DXM to final concentration of 10 nM (3) and porous PCL scaffold with loaded DXM to final concentration of 100 nM (4). Representative measurements of three distinct sets of data have been reported, *indicates *P*-values of <*0.05* for *Student’s t-test*.

In order to assess the onset of the osteoblastic activity of MG63 and the bone forming potential of the cells on non-porous, microporous PCL scaffolds, without DXM and with DXM loaded to a final concentration of 10 nM and 100 nM, the quantification of the alkaline phosphatase enzyme activity according to the p-nitrophenol assay (ALP) was performed (Fig. 9). On microporous PCL scaffolds loaded with DXM to a final concentration of 10 nM and 100 nM seeded after 7–21 days, a considerably higher ALP activity respect to the osteoblasts seeded on the non-porous and microporous PCL scaffold without loaded DXM was seen.

## Conclusion

A new easy strategy to successfully produce bifunctional 3D microporous scaffolds has been developed to mimic the native bone microenvironment and for sustained local release of therapeutic agents, such as dexamethasone which is used as drug model in the cancer medicine regenerative field. Our scaffolds were composed of two key components: (1) a microporous PCL scaffold, obtained from dissolution of polyelectrolyte multilayer coated calcium carbonate microparticles, for cell growth and (2) polyelectrolyte multilayers anchored to PCL pore inner surfaces used as a local sustained DXM release.

Our microporous PCL scaffolds, thanks to their physico-chemical characteristics, also demonstrated that osteoblast-like cells on the functionalized scaffolds promoted good adhesion, proliferation and differentiation in terms of viability and ALP activity of osteoblasts. Furthermore, *in vitro* drug release tests revealed a 43% DXM release faster for pH pathological (acid pH) environments. The release of dexamethasone from nanoscaled polyelectrolyte multilayer’s of CaCO_3_ microspheres might provide a functional pharmacological method to address strategies for cell-based therapies and tissue engineering.

In fact, our work indicates that these biomimetic scaffolds represent a promising candidate for bone tissue regeneration. Changing the therapeutic agent loaded in the scaffolds represents a potential application for sustained local drug release for cancer medicine regenerative, not only for bone cancers but also for the cancer treatment in other body districts.

## Methods

### Materials

All tissue culture media and serum were purchased from Sigma-Aldrich, MG63 cell line was purchased from American Tissue Type Collection (ATTC). The chemicals were supplied by Sigma-Aldrich: poly (allylamine hydrochloride) (PAH), dextran sulfate sodium salt from Leuconostoc spp. (DXS), calcium chloride anhydrous (CaCl_2_), sodium carbonate (Na_2_CO_3_), ethylenediaminetetraacetic acid disodium salt dihidrate, trypan blue, phosphate buffered saline, lysozyme from hen egg white, bicinchoninic acid (BCA) protein assay, bovine serum albumin (BSA), Dexamethasone (DXM), polyacrilammide solution, Metabolic assay, 3-[4,5-dimethylthiazol-2-yl]-2,5-diphenyl tetrazolium bromide (MTT), Trypsin solution, sodium dodecyl sulphate (SDS), phosphate buffer saline (PBS), mouse monoclonal anti-vinculin-FITC, phalloidin-TRICT, DAPI. Alkaline Phosphatase Assay Kit from Abcam. Coomassie Brillant Blue from Biorad.

### Synthesis and characterization of Dexamethasone loaded CaCO3 microparticles

The synthesis of calcium carbonate (CaCO_3_) microparticles and dexamethasone (DXM) loading was performed as described in detail elsewhere^34,36^.

### Synthesis of 3D microporous PCL scaffold

3D microporous PCL scaffolds were obtained using solvent casting and particulate leaching technique (SC/PL). PCL polymer pellets (MW 45,000) were completely dissolved under stirring in chloroform (1:7 w/v) for 3 hours at RT. The PCL solution was added to the CaCO_3_ microparticles (PCL:CaCO_3_ weight ratio 2:3) to form PCL-CaCO_3_ under constant stirring for 30 min. The mixture was sonicated for 5 minutes in ice, and poured into a teflon mould (around 2 cm length, 2 cm width, 1 cm thickness). The obtained mixture was dried at RT overnight, was and subsequently dipped in absolute ethanol for 2 hours at RT, to separate chloroform in a phase inversion process. The PCL/CaCO_3_ microparticles composite was leached in EDTA solution (0.5 M, pH 8.5) overnight to remove the CaCO_3_ microparticles, creating voids into the scaffold. CaCO_3_ microparticles were used as porogen agent, and subsequently leaching, the DXM loaded polyelectrolyte PAH/DXS layers remained connected with electrostatic interaction to PCL scaffolds. The zeta potential of polyelectrolytes and PCL were assessed by Dynamic Light Scattering (DLS) analysis using a Zetasizer Nano ZS90 (Malvern Instruments Ltd., USA) equipped with a 4.0 mW He-Ne laser operating at 633 nm and an avalanche photodiode detector. PCL scaffolds prepared without addition of CaCO_3_ microparticles served as the control. To produce fluorescent PCL scaffolds and ensure the electrostatic interaction of polyelectrolyte with PCL surface, in the coating procedure of CaCO_3_ microparticles was used a solution of fluorescein isothiocyanate-dextran (DXS-FITC, 2 mg/mL with 0.1 M NaCl) indeed DXS solution. Before cell seeding, PCL scaffolds were washed in ethanol and phosphate buffer saline (PBS, 1x) under sterile conditions.

### Scaffold characterizations

#### Scanning electron microscopy (SEM)

The structural morphologies of the porous PCL scaffolds were evaluated using a scanning electron microscope (Carl Zeiss Merlin equipped with Gemini II column and Field Emission Gun - FEG). The scaffolds were freeze-fractured using liquid nitrogen to visualize their inner structure. Prior SEM observation, the scaffold surface was sputter-coated with a 10 nm gold layer to make them electronically conductive and to avoid electronic charging during SEM imaging.

#### Mechanical analysis

Nanomechanical mapping of non-porous and porous PCL scaffolds was performed in PeakForce QNM (PF-QNM) mode^50,51^ on a Bruker MultiMode VIII AFM with controller Nanoscope V at ambient conditions in tapping mode. The samples were investigated using a rectangular silicon cantilever with nominal spring constant of 3 N/m. The cantilever was calibrated on the calibration samples (Bruker, USA) - typically low-density polyethylene and polystyrene with Young’s moduli ranging from 100 MPa to 2 GPa (polyethylene) and from 1 to 20 GPa (polystyrene). The spring constant was measured by a thermal tuning method. The oscillation frequency of the Z-piezo was 1 kHz. The tip radius was obtained by tapping mode imaging of a Bruker reference tip-check sample and then analysed by commercial Nanoscope software. The Young’s modulus, E, is obtained by fitting the unloading curve using the Derjaguin, Muller, Toropov (DMT) model^52^, which takes into account the adhesive force between the tip and the surface. The analysis of the Derjaguin-Mueller-Toporov (DMT) modulus was performed by the software Nanoscope Analysis.

#### Hydration analysis

To evaluate the hydration properties of non-porous and porous PCL scaffolds hydration kinetics and water contact analysis have been performed. Hydration kinetics was assessed in phosphate buffer saline (PBS, 1x) to 37 °C. In particular, the samples were placed into a PBS solution and taken out at specific time intervals. After removal of the excess of surface water, the samples were weighted. The hydration degree was expressed using Equation 1:

1 $$ \% \,{\bf{H}}{\bf{y}}{\bf{d}}{\bf{r}}{\bf{a}}{\bf{t}}{\bf{i}}{\bf{o}}{\bf{n}}\,{\bf{d}}{\bf{e}}{\bf{g}}{\bf{r}}{\bf{e}}{\bf{e}}=\frac{({{w}}_{{t}}-{{w}}_{0})}{{{w}}_{0}}\ast {\bf{100}}$$

where w_t_ was the weight of the sample at fully hydrated state and the w_0_ was the original non-hydrated sample.

To determine surface hydrophilicity, contact angle measurements of non-porous and porous PCL scaffolds were carried out at RT. An OCA20 system from Data Physics was used through the Sessile Drop method. Representative measurements of three distinct sets of data have been reported.

### Protein adsorption

The protein adsorption on PCL scaffolds was determined by soaking the scaffolds in PBS 1x (pH 7.4) overnight and then in 10% fetal bovine serum (FBS) for 24 hours at 37 °C. The scaffolds were washed three times with PBS 1x for 10 minutes to remove non-specifically adsorbed proteins. Then scaffolds were subjected to a 2% sodium dodecyl sulfate (SDS) solution under shaking conditions (100 rpm, 37 °C) for 6 hours to collect adhered proteins. The supernatant was collected and evaluated using bicinchoninic acid (BCA) protein assay reagent (Sigma Aldrich). The BSA was used as a standard and the supernatant was quantified using an UV-visible spectrophotometer (Varian Cary® 300 Scan; Varian Instruments, CA, USA) at a wavelength of 562 nm. Representative measurements of three distinct sets of data have been reported (Student t- test, P < 0.05).

The recovered serum protein samples were subject to fractionation through 4–12% SDS- polyacrylamide gel electrophoresis (SDS-PAGE). The resolved protein bands were visualized by Coomassie brilliant blue staining (BioRad), according to the manufacturer’s instructions. Representative results of three independent experiments have been reported.

### DXM release of PCL scaffolds

Release behavior of DXM from PCL scaffolds was investigated at pH 6.0 (tumor or inflammation environment pH) and pH 7.4 (physiological pH of normal tissue). PCL scaffolds were incubated in a solution of PBS 1x pH 6 or pH 7.4 and kept at 37 °C. At specified time intervals, an amount of release media was removed and the concentration of DXM release was determined from the corresponding absorbance measured in spectrophotometer (Varian Cary® 300 Scan; Varian Instruments, CA, USA) at 260 nm, referring to a standard curve. Samples were taken and analyzed in triplicates. Representative measurements of three distinct sets of data have been reported (*Student t- test*, *P* < *0.05*).

### *In vitro* biological study

Biological assay was performed using human osteoblast-like cell line (MG63) purchased from ATTC. MG63 cells were maintained in DMEM medium supplemented with FBS (10%), 100 units/mL penicillin, 100 μg/ml streptomycin and 2 mM l-glutamine, and sodium pyruvate (5%). Cells were grown in a humidified incubator at 37 °C, with 5% CO_2_ and 95% relative humidity.

#### Viability evaluation

To evaluate cell viability, different assays were performed. The trypan blue assay was performed in accordance to manufacturer’s instructions (Sigma-Aldrich). MG63 osteoblasts were seeded on non-porous and porous PCL scaffolds with or without loaded DXM (50,000 cells/mL, approximately 500 cells/mm^2^) and incubated at 37 °C in 5% CO_2_, 95% relative humidity for 24, 48, 72 hours and for 8 days. The culture medium was changed every 2 days. The viability percentage was expressed using the Equation 2:

2 $${\rm{Cell}}\,\mathrm{viability}\,( \% )=\frac{{\rm{Total}}\,{\rm{viable}}\,\mathrm{cells}\,({\rm{unstained}})}{{\rm{Total}}\,\mathrm{cells}\,({\rm{unstained}}\,{\rm{and}}\,{\rm{stained}})}\ast 100$$

Metabolic assay, 3-[4,5-dimethylthiazol-2-yl]-2,5-diphenyl tetrazolium bromide (MTT) survival tests were performed in accordance to manufacturer’s instructions (Sigma-Aldrich). MG63 osteoblasts were seeded on non-porous and porous PCL scaffolds with or without loaded DXM (50,000 cells/mL, approximately 500 cells/mm^2^) and incubated at 37 °C in 5% CO_2_, 95% relative humidity for 24, 48, 72 hours and for 8 days. The culture medium was changed every 2 days. The absorbance was spectrophotometrically measured at wavelength 570 nm and the background absorbance measured at 690 nm subtracted. The percentage viability is expressed as the relative growth rate (RGR) by Equation 3:

3 $${\rm{RGD}}\,( \% )=\frac{{\rm{Dsample}}}{{\rm{Dcontrol}}}\ast 100$$

where Dsample and Dcontrol are the absorbances of the sample and the negative control. Representative measurements of three distinct sets of data have been reported (*Student t- test*, *P* < *0.05*).

#### Cell morphology

The cell morphology and cell spreading of MG63 osteoblasts on non-porous and porous PCL scaffolds with or without loaded DXM were evaluated by confocal laser scanning microscopy. MG63 osteoblasts were seeded on scaffolds at 50,000 cell/mL (approximately 500 cells/mm^2^ of substrate) in complete culture media, and incubated for 1, 7, 14 and 21 days at 37 °C in 5% CO_2_, 95% relative humidity. At the end of the incubation time, the non-attached cells were removed by rinsing carefully three times with PBS 1x. Focal adhesion contacts (FAC) were detected by cell staining with a monoclonal anti-vinculin-FITC (working dilution of 1:50, Sigma-Aldrich) according the manufacturer’s instructions. Cytoskeleton morphology was investigated by cell staining with phalloidin-TRITC at a final concentration of 1 mg/mL (Sigma-Aldrich), according the manufacturer’s instructions. Nuclei were stained with DAPI (Sigma-Aldrich). Confocal micrographs were taken with a Leica confocal scanning system mounted into a Leica TCS SP5 (Leica Microsystem GmbH, Mannheim, Germany), equipped with a 40x and 63x oil immersion objective and a spatial resolution of approximately 200 nm in x-y and 100 nm in z.

#### Alkaline Phosphatase Assay

The alkaline phosphatase activity of the MG63 osteoblasts on non-porous and porous PCL scaffolds with or without loaded DXM (50,000 cells/mL, approximately 500 cells/mm^2^) was determined for 7, 14, and 21 days of culture using an ALP colorimetric assay kit (Abcam) following the manufacturer’s instructions. ALP activity was calculates by Equation 4:

4 $${\rm{ALP}}\,\mathrm{activity}\,(\frac{{\rm{U}}}{{\rm{mL}}})=\frac{{\rm{A}}}{{\rm{V}}}/{\rm{T}}$$

where A is amount of pNP generated by samples (in μmol), V is volume of sample added in the assay well (in ml) and T is reaction time (in minutes). Representative measurements of three distinct sets of data have been reported (*Student t- test*, *P* < *0.05*).

### Statistical analysis

All experiments were achieved in triplicate. Data are expressed as the mean ± standard error of the mean. Student’s t-test or analysis of variance were performed to compare the means of two or more than three experimental groups, respectively. P-values < 0.05 were considered significant.

### Data availability

The datasets generated and/or analysed during the current study are included in this published article (and its Supplementary Information file) and are available from the corresponding author on reasonable request.

## Electronic supplementary material

SUPPLEMENTARY INFORMATION FOR Therapeutic PCL scaffold for reparation of resected osteosarcoma defect
