# Supplementary material for: Therapeutic PCL scaffold for reparation of resected osteosarcoma defect
Source: Sci Rep. 2017 Oct 4;7:12672. doi: 10.1038/s41598-017-12824-3 (PMC5627265; doi:10.1038/s41598-017-12824-3)
Supplement: Supplementary file 1 — SUPPLEMENTARY INFORMATION FOR Therapeutic PCL scaffold for reparation of resected osteosarcoma defect [file 41598_2017_12824_MOESM1_ESM.pdf]

# SUPPLEMENTARY INFORMATION

# FOR

# Therapeutic PCL scaffold for reparation of resected osteosarcoma defect

**Ilaria E. Palamà[1*], Valentina Arcadio[2], Stefania D'Amone[1], Mariano Biasiucci[3], Giuseppe Gigli[1,4] and Barbara Cortese[2*]**

[1]Nanotechnology Institute, CNR-NANOTEC, via Monteroni, Lecce, 73100, Italy;

[2]Nanotechnology Institute, CNR-NANOTEC, University La Sapienza, P.zle A. Moro, Roma, 00185 Italy;

[3]Center for Life Nano Science@Sapienza, Istituto Italiano di Tecnologia,Viale Regina Elena 291, 00161,Roma, Italy;

[4]Dept. Matematica e Fisica 'Ennio De Giorgi', University of Salento, via Monteroni, Lecce, 73100, Italy

[*]corresponding. ilaria.palama@nanotec.cnr.it, barbara.cortese@nanotec.cnr.it

## Additional Methods

**SEM analysis.** The cell morphology of MG63 osteoblasts were analysed by SEM. The cells were seeded on porous and non-porous PCL scaffolds at 50 000 cells per mL (approximately 500 cells per $mm^2$ of substrate) in complete culture media, and incubated for 7 days at 37 °C in 5% $CO_2$,
95% relative humidity. Subsequently, the non-attached cells were removed by rinsing carefully with PBS 1× at least three times, and fixed with 2.5% glutaraldehyde in PBS 1× for 5 minutes and post fixed with 1% osmium tetroxide for 1 hour in the dark at RT. The fixed cells were washed several times with distilled water and dehydrated in graded ethanol concentrations.
Prior to SEM observation, the samples were sputter-coated with a 10 nm gold layer to make them electronically conductive and to avoid electronic charging during SEM imaging. SEM analyses were taken with a Carl Zeiss Merlin SEM equipped with a Gemini II column and a Field Emission Gun (FEG).

## Additional Figures

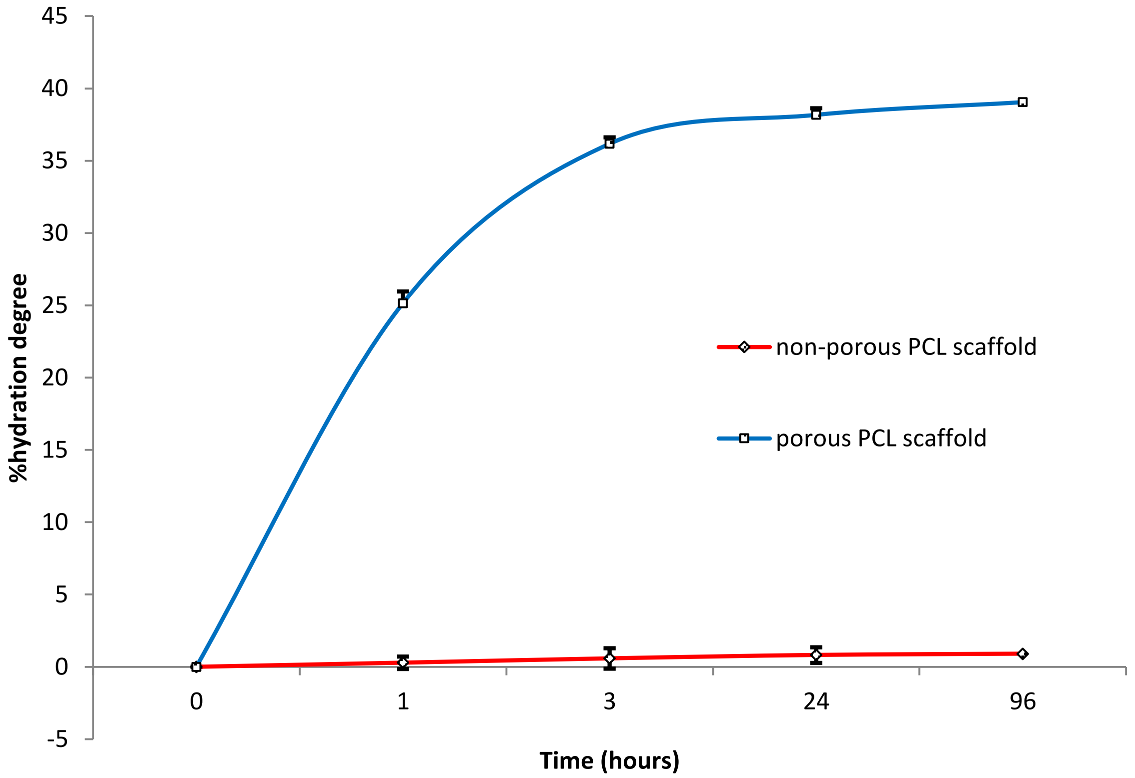


**Figure S1.** Hydration degree (%) to the porous and non-porous PCL scaffolds, after various soaking time in PBS at 37°C. Representative measurements of three distinct sets of data have been reported with no significant difference between values among scaffold groups (*t-Student's test, P<0.05*)

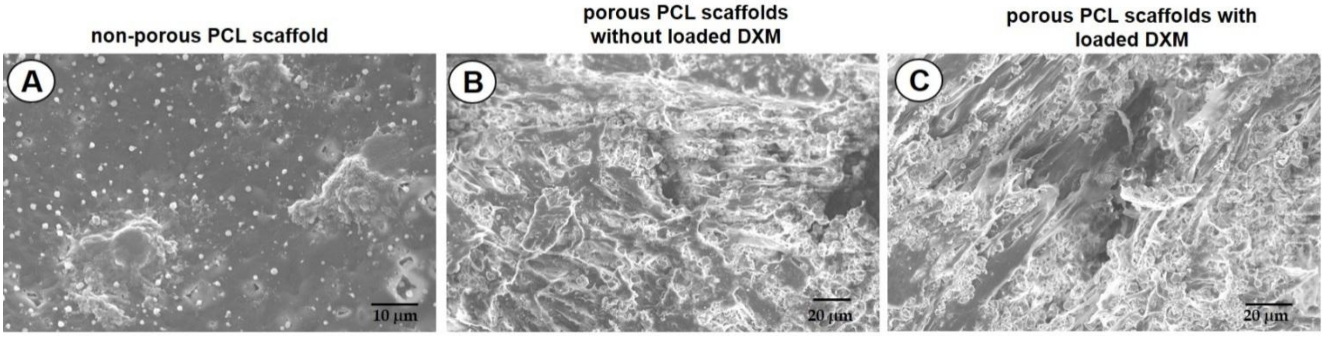


**Figure S2.** SEM images of MG63 osteoblasts seeded on non-porous PCL scaffolds (A), microporous PCL scaffold without (B) or with (C) loaded DXM (final concentration 10 nM) for 7 days. A representative result of three independent experiments is shown. *Scale bars: 10 µm (A), 20 µm (B,C).*

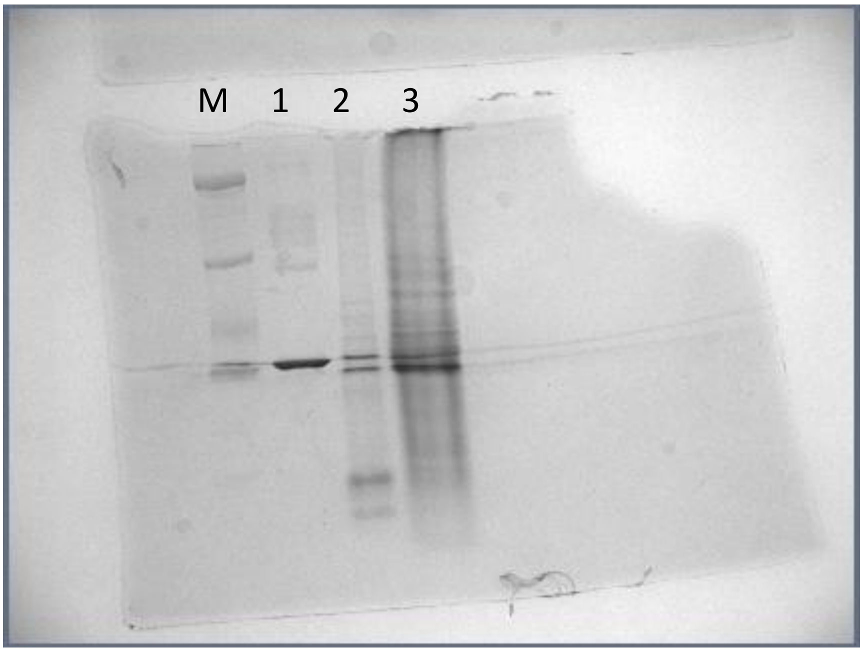


**Figure S3.** Full-length SDS-polyacrylamide gel stained with Coomassie brillant blue,. Lane M, high molecular weight marker; Lane 1, bovine serum proteins; Lane 2, adsorbed bovine serum proteins to the non-porous PCL scaffold; Lane 3, adsorbed bovine serum proteins to the porous PCL scaffold. A representative result of three independent experiments is shown.
